# Emergence of African Swine Fever Virus, Northwestern Iran

**DOI:** 10.3201/eid1612.100378

**Published:** 2010-12

**Authors:** Pooneh Rahimi, Amir Sohrabi, Javad Ashrafihelan, Rosita Edalat, Mehran Alamdari, Mohammadhossein Masoudi, Saied Mostofi, Kayhan Azadmanesh

**Affiliations:** Author affiliations: Pasteur Institute of Iran, Tehran, Iran (P. Rahimi, A. Sohrabi, R. Edalat, K. Azadmanesh);; University of Tabriz, Tabriz, Iran (J. Ashrafihelan);; Veterinary Organization, Tabriz (M. Alamdari, M. Masoudi, S. Mostofi)

**Keywords:** African swine fever virus, wild boars, pathology, PCR, real-time PCR, viruses, Iran, dispatch

## Abstract

In 2008, African swine fever was introduced into Georgia, after which it spread to neighboring Armenia, Azerbaijan, and the Russian Federation. That same year, PCR and sequence analysis identified African swine fever virus in samples from 3 dead female wild boars in northwestern Iran. Wild boars may serve as a reservoir.

African swine fever (ASF) is a notifiable, highly contagious, lethal, hemorrhagic disease in domestic pigs (*1**,**2*). ASF virus (ASFV) (International Committee on Taxonomy of Viruses database no. 00.002.0.01.001), an enveloped double-stranded DNA virus, is the only known DNA arbovirus (*3*). Maintenance and transmission of ASFV involves cycling of virus between soft ticks of the genus *Ornithodoros* and wild pigs (warthogs, bush pigs, and giant forest boars) (*1**,**2*). The virus can also be acquired through ingestion of contaminated feed.

The syndrome caused by ASFV in pigs was initially described in Kenya and later in most other African countries (*1**,**4*). In Africa, it causes a long-term, persistent infection in warthogs and bush pigs (*2**,**3**,**5*). Clinical diagnosis of ASF is difficult because signs of ASF and other hemorrhagic diseases are similar and because virulence varies among ASFV isolates (*1**,**2**,**5**,**6*).

In June 2007, ASFV was identified in the Caucasus region, including Georgia, Russian Federation, and Armenia (*2*). Diagnosis near the port of Poti, Georgia, was based on clinical findings, and virus identification was later confirmed by laboratory investigations. ASFV might have been introduced into Georgia by ships carrying contaminated pork or pork products from other countries. After entering Georgia, the virus extended into Armenia in August 2007. The probable route of virus entry into Armenia was movement of infected pigs and wild boars across the border (*7*). By the end of 2007, an outbreak had occurred in Yerevan and Ararat, after which 1 additional case occurred in February 2008.

In December 2007, the Russian Federation reported its first ASF outbreak since the 1970s. The virus may have entered through neighboring Georgia (*7**,**8*). In January 2008, presence of ASF was officially confirmed in northwest Azerbaijan, ≈180 km east of the Georgia border (village of Nic). Because most residents of Nic keep pigs in backyard smallholdings, ASFV may have entered Nic in contaminated pork (or pork products) or in infected wild boars (*7**,**8*).

In December 2008 and January 2009, ASFV spread to wild boars in northwestern Iran. As in Georgia, initial diagnosis was based on clinical signs and postmortem examinations. Virus identification was subsequently confirmed by laboratory investigations.

## The Study

The wild boar population in northwestern Iran is 12,000–13,000. Boars affected by ASFV show weakness, difficulty walking, dragging of the hind legs, dysentery, and sudden death. The first report of dead boars came from 2 villages (Oshdibin and Namngah) in the Jolfa area and then from other cities such as Ahar, Sarab, Maragheh, and Marand. The disease spread to the city of Khoie, located in another province (West Azarbaijan). Postmortem histopathologic investigations of tissue samples of 3 dead, female, wild boars found characteristic signs of ASF, such as disseminated intravascular coagulation with multiple hemorrhages (Figure).

**Figure:**
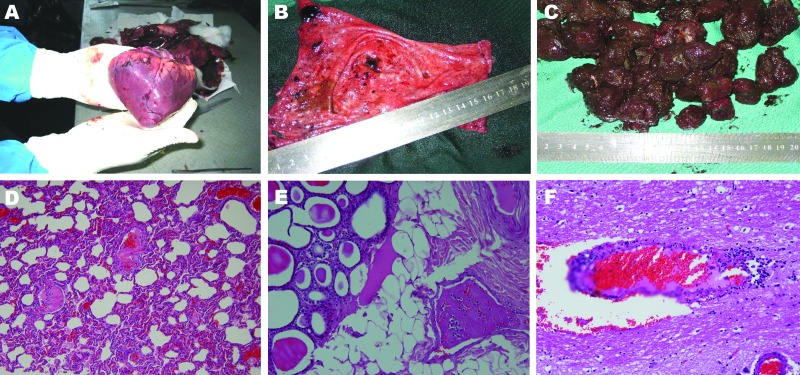
Acute form of African swine fever in wild boars. A) Petechial and larger ecchymotic hemorrhages beneath the epicardium. B) Severe hyperemia and petechial and larger ecchymotic hemorrhages in mucosa of urinary bladder. These hemorrhages are common in acute infectious fever and hemorrhagic diathesis. C) Blood-tinged colon contents with fecal balls covered by thick, blood-stained mucus. D) Congestion and fibrinous thromboses in pulmonary vessels and thickening of alveoli (hematoxylin and eosin stain; original magnification ×100). E) Fibrinous thrombus in a venule within interlobular adipose tissue of the thyroid gland (hematoxylin and eosin stain; original magnification ×200). F) Blood vessel congestion, perivascular hemorrhage, lymphocytic perivascular cuffing, and infiltration with degenerating lymphocytes (hematoxylin and eosin stain; original magnification ×200).

Viral DNA from 6 tissue samples (kidney, liver, lung, large intestine, heart, and spleen) from the 3 dead boars was extracted by using the JETQUICK Tissue DNA Spin Kit (GENOMED GmbH, Lohne, Germany) according to the manufacturer’s instructions. ASFV was detected by PCR and SYBR Green real-time PCR on a Rotor-Gene 65H0 (Corbett Life Sciences, Sydney, New South Wales, Australia) in all samples from each boar; primers used were P72 D, U (major capsid protein), and PPA_1,2_ (in the viral protein 73 coding region of the genome) (*6**,**9*). Melting curve analysis showed that an elevated temperature of 86.7°C could generate a specific peak in this curve. The sequences of the PCR products derived by using PPA primer pairs were analyzed by using BLAST (www.ncbi.nlm.nih.gov/blast/Blast.cgi) and showed 100% similarity to submitted sequences of Georgia isolates. A partial sequence of the isolates from Iran has been submitted to GenBank under accession no. FJ897727.

## Conclusions

To confirm a specific pathogen and trace the possible sources of infection, genetic characterization of the virus strain associated with disease is crucial (*1**,**2*). Real-time PCR and PCR are the most practical ways to differentiate infectious agents that cause similar clinical signs (*5**,**6**,**9*). The 100% similarity between our isolates and those from Georgia suggests that they might have originated from Georgia, probably brought into Iran by infected wild boars crossing the Aras River during the disease incubation period.

The main obstacles to ASF eradication are wildlife reservoirs, limited ability to control movement of infected pigs and wild boars, inadequate laboratory support for rapid and accurate diagnosis, and lack of an effective vaccine (*4*). *Ornithodoros* spp. ticks may contribute to virus persistence in the Caucasus region, including northwestern Iran. Although in our study ticks were not detected on the boar carcasses, they should be considered as potential transmission vectors. *O. lahorensis* ticks are found in areas with a cold climate and are mainly found near sheep and cows, so they are not likely to be isolated from an animal. In the absence of *Ornithodoros* spp. ticks, transmission to pigs could occur through contact with infected pigs or through feeding of virus-contaminated products (*7**,**8*).

The pig industry differs among Caucasus countries. In Azerbaijan, Chechnya, and southern Russian Federation, the pig industry is not as large as it is in Georgia and Armenia (*7*). In these countries with limited or no pig farms, the virus could be spread by infected wild boars, and the disease could become endemic as it has in Spain and Sardinia (*3**,**7**,**8**,**10**–**12*). Because Iran has no commercial pig facilities, the ASFV reservoir would be wild boars, which could transmit disease to neighboring countries that have pig industries, resulting in considerable economic losses.
